# Accelerating start-up cycles in investigator-initiated multicenter clinical trials

**DOI:** 10.1017/cts.2025.10180

**Published:** 2025-10-24

**Authors:** Shannon Hillery, Ryan Majkowski, Ying Wang, Bradley Barney, Lindsay Eyzaguirre, Andrew Mould, Nichol McBee, Esther Woo, Elizabeth Holthouse, Kenneth Wiley, Salina P. Waddy, Daniel Ford, Daniel F. Hanley, Karen Lane

**Affiliations:** 1 Department of Neurology, BIOS Clinical Trial Coordinating Center, Trial Innovation Center, Johns Hopkins University School of Medicine, Baltimore, MD, USA; 2 Yale Center for Clinical Investigation, Yale University School of Medicine, New Haven, CT, USA; 3 School of Medicine and Utah Data Coordinating Center, University of Utah, Salt Lake City, UT, USA; 4 National Center for Advancing Translational Sciences, Bethesda, MD, USA; 5 Institute for Clinical and Translational Research, Johns Hopkins University, Baltimore, MD, USA

**Keywords:** Multicenter clinical trials, clinical trial start-up, team science, lean workflow pathways, trial innovation network

## Abstract

**Background:**

Operational roadblocks and organizational delays in multicenter clinical trials have been evident for decades, with the start-up cycle being especially notorious for setbacks. To address these challenges and improve multicenter clinical trial execution, we developed an accelerated start-up (ASU) management strategy – a structured site onboarding approach based on lean management principles.

**Methods:**

Three elements were integrated into the strategy: a standardized workflow, a dedicated site navigator (SN), and an electronic tracking system. We examined the range, central tendencies, and distribution of site activation times among differing combinations of these three elements. To determine how these combinations affected individual start-up milestones, we fit mixed models to compare percent achievement of predetermined milestone benchmarks and time to completion.

**Results:**

Thirteen consecutive trials (*n* = 308 site activations) employed three distinct combinations of the three ASU elements. Trials using all three elements (*n* = 6) had 160 total site activations in a median of 133 days. Three trials without the SN element had 52 total site activations in a median of 191 days. Four trials without the standardized workflow element had 96 total site activations in a median of 277 days. Significant differences between combinations included times to sIRB submission (*p* = 0.004), training/certificates completion (*p* = 0.03), and site activation (*p* = 0.003). Results suggest sites activated faster and achieved predetermined benchmarks for every milestone more often when three elements were employed.

**Conclusion:**

This sample trial start-up data supports that sites can meet ambitious timelines, underscoring the strategy’s potential to streamline workflows and improve site team performance.

## Introduction

Operational roadblocks and organizational delays in multicenter clinical trials have been evident for decades [1,2]. There is a need to find ways to reduce delays and improve clinical trial timelines. Industry continues to describe start-up cycle times much the same as they were 20 years ago, reporting more than two months to select sites, then another eight months from site selection to site activation [3,4]. In the world of clinical trials, trial designs are becoming more complex, and trials are becoming larger, inevitably increasing the time and cost it takes to plan, start-up, and conduct a clinical trial [5]. Much has been written regarding why clinical trials are not completed on time, citing delays in site start-up among the top causes.

In 2016, the National Institutes of Health’s (NIH) National Center for Advancing Translational Sciences (NCATS) formed the Trial Innovation Network (TIN) to increase the efficiency and effectiveness of multicenter clinical trials by focusing on innovation and collaboration and by leveraging the strength and expertise of the Clinical and Translational Science Award (CTSA) Program [6,7]. The TIN is an inter-institutional consortium comprised of four key partners: NCATS, CTSA Program hubs, multiple Trial Innovation Centers (TICs), and a trial Recruitment Innovation Center (RIC) [8]. Innovations have included how to coordinate with a single institutional review board (sIRB) [9,10], contract streamlining [11], advancements in site selection and feasibility assessments [12], and technologies to build social gaming for site start-up engagement [13]. An important innovation being assessed is a site-centered accelerated start-up (ASU) management strategy – a structured onboarding of sites, with defined operational methods based on lean management principles championed by the manufacturing industry.

**Figure 1.**
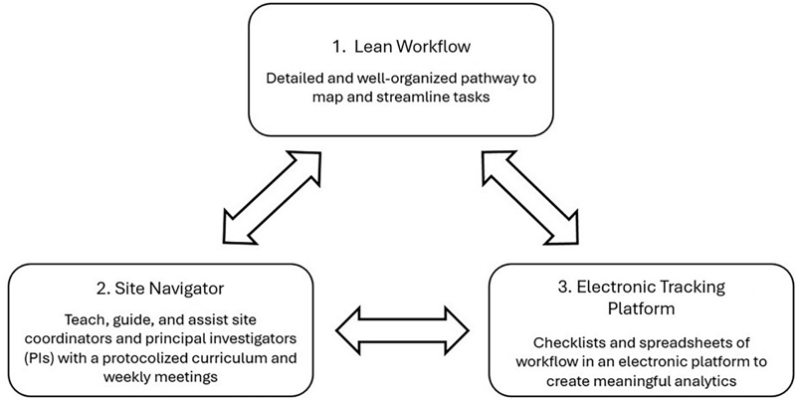
Three Elements of the Accelerated Start-Up (ASU). The three elements unify a management strategy for activating sites more rapidly and efficiently in multicenter clinical trials. The three elements are: a workflow, utilizing lean management principles to map a standardized start-up sequence with milestones benchmarked against a specified timeline; a dedicated site navigator (SN) to guide sites through the workflow, using a corresponding training curriculum at mandatory one-on-one weekly meetings with each site team; and a tracking system (preferably electronic) automating the workflow to measure progress.

As a management strategy, the ASU integrates three elements: (1) a workflow, utilizing lean management principles, that maps the leanest start-up sequence with milestones benchmarked against a standardized, prescribed timeline; (2) a dedicated site navigator (SN) to guide sites through the workflow, using a corresponding training curriculum presented at mandatory one-on-one weekly meetings with each site team; and (3) a tracking system automating the workflow to manage resources effectively and measure site team progress (Figure 1).

We describe an exploratory analysis of the impact of the ASU strategy on start-up timelines for 13 consecutive multicenter trials. We examined the range, central tendencies, and distribution of site activation times, and how using different elements of the ASU strategy might affect site start-up timelines.

## Methods

### Defining the ASU strategy

Lean management is a philosophy developed in the 1980s by Japanese automotive manufacturers [14]. Paramount to managing lean is creating workflow pathways that flow continuously and effortlessly in a proper sequential or parallel order. Lean management is a process of aligning milestones in such a way that a worker’s tasks are accomplished with a minimum of “waste,” achieved by eliminating inefficiencies such as setbacks from production out of sequence, duplicative work, unnecessary down time, and mistakes. Once the leanest pathway is found, the workflow sequence can be standardized. Standardization then leads to uniform milestone execution, and teams are taught the same way every time on how to manage the production line, thereby increasing operational (quality and timeliness) performance [15].

We systematically reorganized start-up tasks more effectively and without interruption into parallel task-completion sequences required for sIRB approval, contract execution, delegation of responsibility, regulatory document collection, and training on the protocol. Three parallel pathways were created: (1) a pathway for the investigative team to complete a delegation log, collect regulatory documents, and train on the protocol; (2) an institutional review board (IRB) pathway to obtain site HRPP (Human Research Protection Program) reliance and local approvals and then for sIRB officials to approve the team as a site and the consent materials; and (3) a contract pathway for local and sponsoring contract officials to execute site subcontracts.

**Figure 2.**
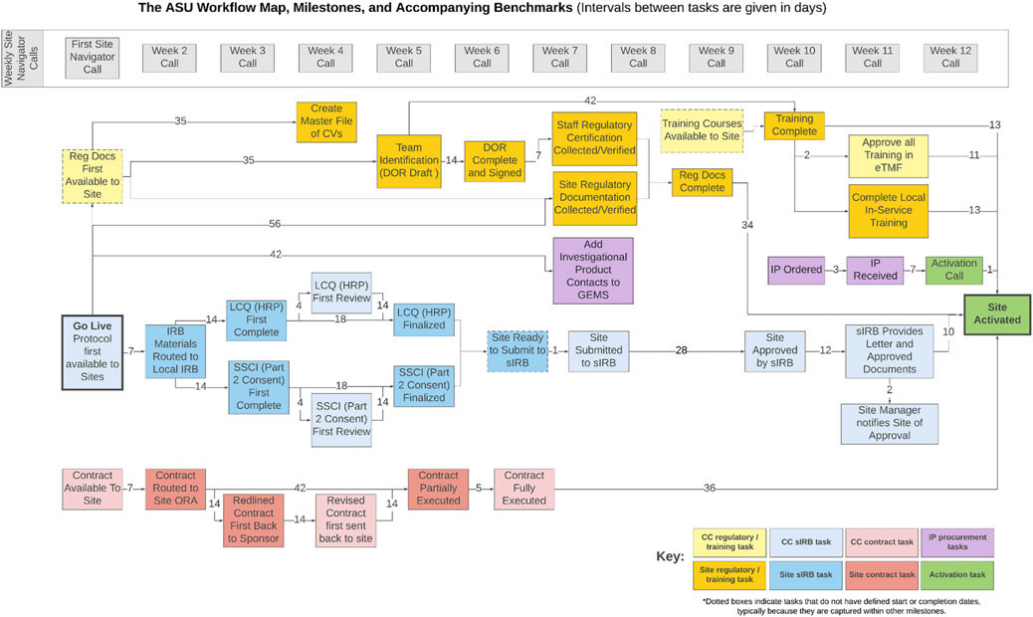
The ASU workflow map, milestones, and accompanying benchmarks. Three parallel pathways, regulatory and training (yellow), sIRB approval (blue), and contract execution (pink), have a specifically ordered workflow in the leanest sequence with prescribed benchmarks (timelines). Benchmarks for tasks are defined by numbers (in days) on interval lines between tasks. Task responsibility is indicated by the shade: darker boxes indicate site responsibility; lighter boxes, coordinating center responsibility. Dotted boxes indicate tasks that do not have defined start or completion dates, typically because they are captured within other milestones. This example is mapped for a 90-day start-up goal. Weekly meetings, led by site navigators (SNs) with set agendas and presentations via a slide-deck curriculum, are depicted as gray boxes along the top of the map. Abbreviations: CV = curriculum vitae; DOR = delegation of responsibilities; eTMF = electronic trial master file; GEMS = global electronic management system; HRP = human research protections; IP = investigational product; IRB = institutional review board; LCQ = local context questionnaire; ORA = office of research administration; sIRB = single institutional review board; SSCI = site-specific consent information.

Once the sequence for the streamlined, standard workflow pathways was decided, pragmatic and achievable benchmarks were needed to add timelines to the start-up steps. In 2017, the Tufts Center for the Study of Drug Development (CSDD) completed a start-up metrics demonstration project at the same time as the ASU strategy was in development. This TIC-supported metrics project benchmarked various start-up tasks based on data from a series of federally funded investigator-initiated trials, 10 industry trials, and two Phase III trials previously managed by the ASU development team. The results (unpublished data) signaled 90 to 120 days could be a leaner goal for a site to activate, as a reasonable portion of both the federally funded and industry trials activated within the same 120-day time limit as the two previous Phase III trials managed by the ASU team. Using average timeline results for individual tasks from the metrics project, benchmarks were assigned for the key milestones in each of the three workflow pathways. The pathways and benchmarks were then combined to create a workflow map of sequential, one-step-at-a-time, time-sensitive milestones with timeline expectations quantified for each step that, when added together, approximate a 90-day activation period (Figure 2).

### Automating the workflow

The workflow map was built into an interactive platform, Global Electronic Management System (GEMS, Prelude, Austin, TX). GEMS was programmed to calculate and populate fields with expected completion dates to match the predetermined benchmarks mapped for each task, enabling an automated display of real-time deadlines for tasks and milestones. Fields for actual completion dates were then created to track when each site milestone was achieved. Interactive features were programmed into the system to give credit for milestones delivered on time and warnings for upcoming deadlines and overdue work, so site navigators could use both calculated benchmark dates and interactive features to drive progress and recognize problems. From 2017 to 2018, we tested the GEMS tool in three trials. The testing also coincided with the new Common Rule [16] mandating single IRBs for the first time. The GEMS platform, operation manuals, and the content for the weekly meetings were adjusted to better represent the intricacies of reliance on a single IRB.

### GEMS pathways and benchmarks as an automated standard operating procedure (SOP)

GEMS functioned as a step-by-step ASU standard operating procedure (SOP) for both site teams and navigators to follow. Once a start date was programmed into GEMS, the expected 90-day activation date and due dates for sequenced tasks along the parallel pathways were displayed in the same manner for that trial’s entire cohort of sites for the duration of the start-up cycle. Guidelines for using GEMS as an SOP were developed for site principal investigators (PIs) and coordinators to be used during site initiation webinars, and specific parts of the SOP and guidelines were replicated into slide decks to be presented as 12 weekly slide presentations.

### Site navigation

The pre-programmed slide decks were developed to guide SNs and investigative teams through 12 mandatory weekly SN-coordinator meetings to review the workflow and milestones due, as displayed in GEMS. The slide deck discussions matched each week’s expected deliverables and acted as a checkpoint for the next set of deliverables. The SNs were trained to coach and educate site teams on how to set up the trial within their own institutions and share the workflow sequence and benchmarks with local personnel. In addition, SNs were trained to monitor task completion and enter completion dates into the GEMS tracking tool. Instruction templates were developed for SNs to use between weekly meetings to avoid site downtime. SNs entered actual dates of milestones met for every site in all 13 trials to track site progress towards activation and to accrue metrics for performance comparisons.

### Standardization of start-up within each trial

The start-up benchmarks and activation goal of 90 days were preset in GEMS and all other ASU materials prior to the sampling of the 13 trials and have been programmatic since ASU inception. Although trial leadership committees could not change the overall activation benchmark or the individual task benchmarks that aligned with the overall start-up time, they were able to determine how the elements of each start-up would be organized (i.e., full use of all elements, yes/no; SN, yes/no; and lean workflow, yes/no). Once the elements were determined for each trial, every site team in that trial was given the same training module and manual of instructions, the same GEMS SOP, and the same prescribed benchmarks and activation goal. There was no self-selection by a site regarding choice of start-up method.

### Convenience sampling of 13 consecutive trials

We acted as the start-up coordinating center for 13 consecutive trials between May 2019 and August 2024 and tracked benchmarked milestones and actual timelines in the GEMS automated tracking system. As defined in Figure 2, time spent in start-up (in days) was measured as the time between the protocol and IRB packet arrival at the site (“Go Live”) and the site becoming active for enrollment (“Site Activated”). For each trial, we calculated the mean and median number of days and interquartile ranges (IQR) required to complete the trial start-up cycles. We also compared individual milestone completion times to predetermined benchmarks to determine the percentage of sites that met milestone benchmarks.

### Statistical methods

**Figure 3.**
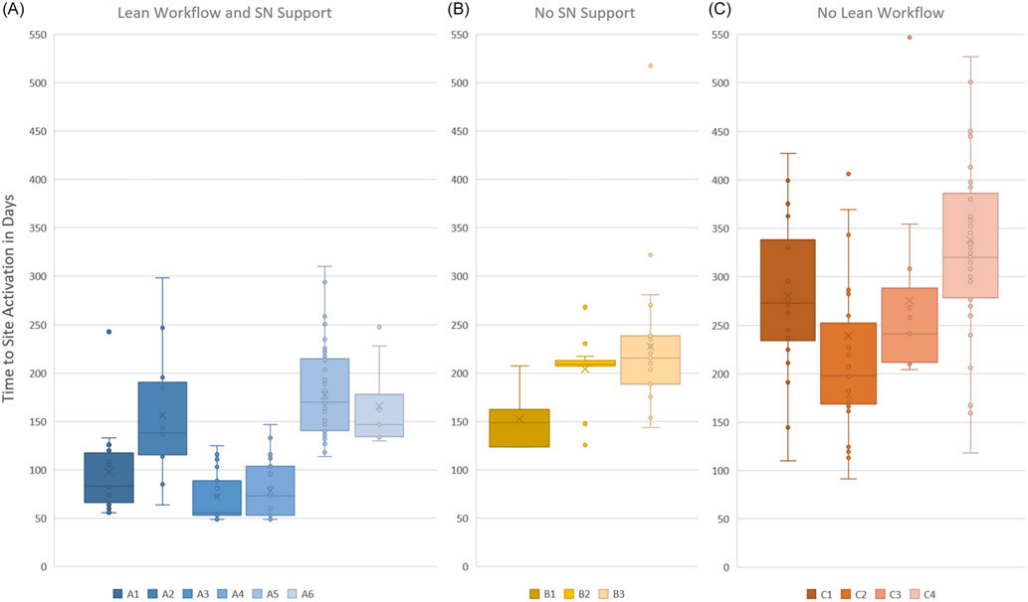
Box and whisker plots displaying site activation times for 13 trials. The distribution of site activation times (in days) as measured from protocol availability to site activation for enrollment (“go live”). The ABC panels are grouped according to which of the three accelerated start-up (ASU) elements each trial utilized: in panel A, six trials (including two COVID-19 trials) utilized all three elements; in panel B, three trials did not utilize dedicated SNs due to personnel shortages; and in panel C, four trials had sponsors who chose not to use a lean workflow, preferring their sites set their own pace and sequence. For display purposes, site activation durations exceeding 550 days (*n* = 3) are not shown in panel C. All 13 trials were tracked using the GEMS platform. Abbreviation: SN = site navigator.

Each trial was categorized according to which combination of the three ASU elements was put into operation for the trial’s full site cohort. The 13 trials combined the elements in one of three ways: all three ASU elements (lean workflow, SN, automated workflow tracking), categorized as Panel A; lean workflow and automatic workflow tracking without SNs, categorized as Panel B; and SN coordination and automated tracking but no utilization of the lean workflow, categorized as Panel C (Figure 3). We examined the effect that each of these combinations had on site activation performance. All 13 consecutive trials were tracked in GEMS regardless of element combinations.

We also calculated the percentage of sites that met predetermined benchmarks for individual milestones. Negative or missing data points were excluded. Institutions may have been involved in more than one trial, but each activation and associated milestone completion durations were regarded as independent between trials as the site teams were generally unique within an institution. We fitted a generalized linear mixed model (GLMM) to model the odds of reaching individual milestone benchmarks, with panels as the fixed effect and with random intercepts for trials (to account for clustering due to trial-specific variation), testing whether the odds were associated with the panel. We also summarized the median and first and third quartiles of durations across panels and tested for differences between panels by fitting a linear mixed model (LMM) to the rank of each site milestone completion time; the rank was used to mitigate the effect of outliers and skewness. Although we also fitted Cox frailty models to assess differences in time-to-event outcomes between panels while allowing for within-study clustering of site-level performance, we do not report the Cox results because of concerns that the proportional hazards assumption would not be reasonable. As a sensitivity analysis, we repeated analyses excluding the two COVID-19 treatment trials that implemented the three ASU elements in a hyper-accelerated public health crisis context, the results of which are included in the supplemental materials. Each test was conducted at alpha = 0.05.

## Results

Central coordinating center fidelity to data entry into the GEMS tracking platform yielded start-up metrics for more than 300 sites participating in 13 multicenter clinical trials. Site teams were recruited from established specialty networks and NCATS CTSA Hubs pre- and post-grant awards and selected according to the study’s needs. The distribution of time to site activation is shown in Figure 3, grouped into three panels according to how many ASU elements were utilized trial-wide during the start-up period, as follows: six trials followed the three ASU elements (Panel A), three trials were not able to employ a dedicated SN element (Panel B), and four chose to not utilize the lean workflow element (Panel C).

In Panel A, six trials activated sites following the three ASU elements. Sites in these trials were guided by dedicated site navigators through the lean workflow and met weekly to review slide decks and track site progress within the GEMS platform. The six Panel A trials achieved a median time to site activation ranging from 56 to 170 days. Overall, these six trials, using all the ASU elements, activated a total of 160 sites in a median of 133 days. The three Panel B trials activated sites following two of the ASU elements. These trials achieved a median time to site activation ranging from 149 to 216 days. Overall, these three trials, without the help of SNs, activated a total of 52 sites in a median of 191 days. The four Panel C trials activated sites utilizing two of the ASU elements. These trials achieved a median time to site activation ranging from 198 to 320 days. Overall, these four trials, without the lean workflow sequencing or benchmarks, activated a total of 96 sites in a median of 277 days, the longest of the three groups. Panel C sites worked with site navigators, but the navigators were directed by trial leaders to not use the prescribed lean workflow sequence or benchmarks, as these study PIs preferred to let site teams work at their own pace and on their own terms. Trial characteristics, the ASU elements employed, and the mean, median, and interquartile range of site activation times for each trial in Figure 3 are presented in Table 1.

**Table 1.** Trial and start-up characteristics

Figure 3 trial label	Therapeutic area and primary network, if applicable	Start-up M/Y	Number of sites activated^*^	Lean workflow	SN support	Electronic tracking	Site activation mean (Days)	Site activation median (Days)	Site activation IQR (Days)
A1	Critically Ill Children (HEAL Pain ERN)	May-2019	18	Yes	Yes	Yes	98	84	67–118
A2	Fetal Renal Anhydramnios (NAFNET)	May-2020	11	Yes	Yes	Yes	157	138	116–191
A3	COVID-19 (CTSA Hubs)	May-2020	25	Yes	Yes	Yes	72	56	53–89
A4	COVID-19 (CTSA Hubs)	May-2020	25	Yes	Yes	Yes	78	73	53–104
A5	Atrial Fibrillation (CTSA Hubs)	May-2023	73	Yes	Yes	Yes	177	170	141–215
A6	Sepsis	Sep-2023	8	Yes	Yes	Yes	166	147	135–179
B1	Ischemic stroke	Dec-2020	19	Yes	No	Yes	153	149	124–163
B2	Pediatric pulmonary HTN (PPHNet)	Jan-2022	12	Yes	No	Yes	205	210	208–214
B3	Normal pressure hydrocephalus (AHCRN)	Mar-2022	21	Yes	No	Yes	228	216	189–239
C1	Alzheimer’s dementia	Jul-2020	20	No	Yes	Yes	280	272	234–338
C2	Osteoarthritic knee pain (HEAL Pain ERN)	Aug-2020	26	No	Yes	Yes	239	198	169–252
C3	Intracerebral hemorrhage (MISTIE III network)	Jan-2022	11	No	Yes	Yes	275	241	212–288
C4	Intracerebral Hemorrhage (MISTIE III network)	Jun-2022	39	No	Yes	Yes	338	320	278–386

Trial and start-up characteristics (organized by Figure 3 ABC panels) comparing utilization of the three ASU elements and the resulting mean, median, and interquartile range of site activation times.

* Excludes sites with a negative time-to-event duration or with missing dates such that a duration could not be calculated.

Abbreviations: HEAL Pain ERN = Helping to End Addiction Long-term Initiative Pain Management Effectiveness Research Network; NAFTNET = North American Fetal Therapy Network; HTN = hypertension; PPHNet = Pediatric Pulmonary Hypertension Network; AHCRN = Adult Hydrocephalus Clinical Research Network; MISTIE = Minimally Invasive Surgery with Thrombolysis in Intracerebral hemorrhage Evacuation; SN = Site Navigator; *M* = month; *Y* = year; SN = Site Navigator; IQR = interquartile range.

Table 2 summarizes, by panel, the percentage of site teams achieving individual predetermined benchmarks during the start-up period. As in Table 3, the total number of start-up instances referred to in the header row represent site activations across the 13 trials. Panel A site teams used all elements and achieved the predetermined benchmarks for every milestone more often than both Panel B (no SN support) and Panel C (no lean workflow) groups. Sites in Panels B and C did not meet the activation benchmark of 90 days, while 31% of sites (49/160 sites) in Panel A were activated in 90 days or less. The GLMM analysis yielded significant differences in achieving the benchmarks for two milestones: sIRB submission (*p* = 0.001) and completing the delegation of responsibility log (*p* = 0.02). Compared to the reference level (implementing all ASU elements), teams using only parts of the ASU program were less likely to achieve the benchmarks, as evidenced by odds ratios less than 1. There were no statistically significant differences for three milestones: contract partial execution, upload of all site-wide regulatory documents, and upload of personnel regulatory documents. For two milestones, training and certificates and activation to enrollment phase, the GLMM estimation had convergence problems (at least one of the element combinations had no sites meeting the benchmark).

**Table 2.** Percent of site teams achieving pre-determined benchmarks

		Milestone benchmark achieved (%)					Odds ratio (95% CI)			
**Start-up milestone**	**Predetermined benchmark (# days from “Go Live”)**	Figure 3 **, Panel A sites** *(Lean workflow & SN support)* ***n* = 160**	Figure 3 **, Panel B Sites** *(No SN support)* ***n* = 52**			Figure 3, **Panel C sites** *(No lean workflow)* ***n* = 96**	Figure 3 **, Panel A sites** *(Lean workflow & SN Support)* ***n* = 160**	Figure 3 **, Panel B sites** *(No SN support)* ***n* = 52**	Figure 3 *(No Lean workflow)* ***n* = 96**	***P*-value** ^*^
sIRB submission	40	44%		6%	7%		Reference	0.08 (0.01, 0.38)	0.09 (0.02, 0.30)	0.001
Contract partial execution	49	69%		31%	31%		Reference	0.08 (0.01, 0.82)	0.14 (0.01, 1.12)	0.06
Delegation of responsibility log	49	33%		2%	6%		Reference	0.02 (0.00, 0.48)	0.03 (0.00, 0.57)	0.02
Regulatory documents	56	59%		4%	28%		Reference	0.01 (0.00, 0.55)	0.37 (0.01, 10.2)	0.08
Personnel regulatory documents	56	58%		4%	21%		Reference	0.02 (0.00, 0.54)	0.31 (0.01, 5.45)	0.08
Training & certificates	77	31%		0%	2%		Not reported – convergence issues in model estimation			
Activation to enrollment phase	90	31%		0%	0%		Not reported – convergence issues in model estimation			

*Notes*: The percentage of site teams achieving seven key pre-determined benchmarks, by ABC combination, and the odds of executing benchmarks (dichotomized success/failure) based on Generalized Linear Mixed Models (GLMMs).

Excludes sites with a negative time-to-event duration or with missing dates such that a duration could not be calculated. Column header site counts reflect the number of sites activated. Denominator per metric may be slightly different.

* *P*-values from logistic regression models with random intercepts for the trial.

Abbreviations: CI = confidence interval; sIRB = single institutional review board; SN = Site navigator.

**Table 3.** Analyses of site team time to completion of key milestones

	Median completion times (Q1, Q3), in days			
**Start-up milestone**	Figure 3 **, Panel A sites** **Lean workflow and SN support**	Figure 3 **, Panel B sites** **No SN support**	Figure 3 **, Panel C sites** **No lean workflow**	***P*-value** ^*^
sIRB submission	48 (22, 84)	91 (63, 115)	96 (70, 190)	0.004
Contract partial execution	32 (16, 64)	77 (46, 106)	89 (37, 147)	0.12
Delegation of responsibility log	58 (45, 80)	112 (89, 139)	211 (84, 296)	0.02
Regulatory documents	50 (33, 73)	126 (96, 150)	108 (54, 235)	0.14
Personnel regulatory documents	51 (39, 81)	119 (89, 148)	154 (64, 243)	0.07
Training & certificates	121 (55, 154)	152 (133, 199)	219 (123, 312)	0.03
Activation to enrollment phase	133 (82, 176)	191 (153, 217)	277 (211, 347)	0.003

*Notes*: Organized by Figure 3 ABC panels, analysis of site team time to completion of seven key milestones based on Linear Mixed Models (LMMs).

Excludes sites with a negative time-to-event duration or with missing dates such that a duration could not be calculated.

* *P*-value from linear mixed model using the ranked event duration as the outcome with random intercepts for trial.

Abbreviations: *Q* = quartile; sIRB = single institutional review board; SN = site navigator.

A LMM analysis for ranked milestone completion performance of each site’s activation (fastest activation = 1, slowest activation = 308) is presented as Table 3. (See Supplemental Figure 1 for a visual display of this performance rank highlighting the relative performance differences across trials.) The LMM demonstrated similar significant effects to the GLMM for benchmark achievement. There were significant differences between panels in time to sIRB submission (*p* = 0.004) and time to having the delegation of responsibility log finalized and signed (*p* = 0.02). The LMM also detected significant differences for the training and certificates (*p* = 0.03) and activation to enrollment phase (*p* = 0.003) milestones, the two milestones that had convergence problems in the GLMM. Trials implementing the three ASU elements (i.e., Panel A) tended to have sites complete milestones in the shortest time.

The COVID-19 pandemic was a unique environmental change. Detailed results of several sensitivity analyses are presented in the supplement materials. There were fewer significant differences when the sensitivity analysis excluded the two COVID-19 treatment trials, but two outcomes retained statistical significance: sIRB submission (for the GLMM and ranked time LMM) and site activation (for the ranked time LMM).

## Discussion

This convenience sample of 13 consecutive trials demonstrates the potential value of the structured lean management strategy that utilized defined pathways, weekly site navigation, and a performance-guiding electronic platform to accelerate site activations in this group of multicenter academic clinical trials.

The start-up tasks were not different; they were strategically reorganized. What was new and unique was requiring that tasks be completed in parallel with precise sequencing and predetermined delivery dates. When the three ASU elements were successfully combined, site teams were able to activate in less time when they were made aware of benchmarks, taught a more efficient organization of work, and ushered through the start-up activities by an experienced start-up navigator and an electronic tracking system operating as a standardized SOP.

Site navigators, with full-time effort reserved for site management, can improve time management at the sites with constant, positive engagement and keep teams on track by simultaneously solving issues as they arise [17]. The idea that site navigation is critical has been recently observed in the increasing numbers of site managers at large clinical research organizations (CROs); however, our approach to how the site navigator works and the tools used is what separates the ASU site navigator role from the rest. Other academic research organizations (AROs) and CROs utilize site navigators but, to our knowledge, not in conjunction with compulsory parallel pathways, a structured program of 12 weekly meetings, and an automated tracking system that maps out the series of tasks in a specific workflow sequence and compares performance against expected benchmarks; nor do other AROs and CRO’s mandate strict adherence to the specific ASU parallel workflow sequence, benchmarks, and weekly reviews of progress mapped in Figure 3. Most of our site teams across all trials have expressed that the ASU program was their first experience of employing an accelerated start-up program structured such as ours.

SNs were trained to understand the steps of the parallel work pathways, the timing of the benchmarks, and how to document team progress with a watchful eye. Persistent SN engagement at weekly meetings and displays of task progress in GEMS helped motivate site teams to not place start-up work on the backburner or shift priorities elsewhere but instead accept deadlines and complete tasks on time. We observe in Table 2 that not having a SN was the panel that typically had the lowest percentages of sites achieving individual milestone benchmarks and had no sites achieving the 90-day activation goal. This suggests that consistent guidance and site team awareness of actual progress against benchmarking are essential to achieving better start-up times. The possibility of a 90-day site start-up appears less feasible without SNs using progress data at weekly meetings to engage and motivate sites to stay the course.

Site navigators come from various experiences and backgrounds, such as research nursing, coordinating center and data center management, clinical research coordination, or from HRPP/Office of Research Administration (ORA) offices. Clinical research associates (CRAs) or in-house coordinating center managers can be assigned as SNs, but during an active start-up cycle, we have learned that the SN role should be assigned as a full-time equivalent (FTE) position for any trial with 10 or more sites and to limit the number of sites for a single navigator.

The start-up cycle is especially notorious for delays due to inefficiencies in project management, both at the trial level and site level. Specific site factors that delay site activation relate to regulatory approval backlogs, training modules put on the back burner and not available to sites when needed, and errors in documentation requiring backtracking for correction. But a significant amount of time is also lost by site teams who do not understand, from the start, that they have benchmarks as deadlines to meet or recognize the leanest pathways to accurately organize their own work to meet those benchmarks [18]. This can lead to production pauses or even work stoppages at the site. When work breaks or stoppages are exceptionally time-consuming, there can be a loss of momentum among investigators, and, in a typical five-year trial funding cycle, this could diminish recruitment time at a site by as much as 10% [19].

In Table 3, the difference of median time to activation between panels B and C suggests that the presence or absence of the lean workflow sequencing, regardless of SN presence, also impacted time to activation. In Panel B, sites were provided with the lean workflow and performance expectations as a checklist and, despite not having a SN to guide the execution of start-up milestones, activated quicker than the site teams in Panel C who had SNs but did not utilize the lean workflow as standard procedure.

Delays are often attributed to site contracting and ethical reviews [18]. The ASU milestones included delivery dates for institutional support items that aligned with the 90-day workflow. Within our analysis, we found benchmarks were met more often along the IRB and contracts pathways (IRB review, partial contract execution) than for the pathway that was solely the responsibility of the site team (regulatory documents, delegation logs, and training certifications); however, these observations are based on merged site team and institutional support unit performance, as internal support unit workflows were not tracked. The GEMS system prompted site teams to work with institutional personnel at their local IRB and contracts office as their first tasks, and the early introduction of due dates as benchmarks and milestones for both institutional and investigative team performance may have improved group awareness of trial timelines. For support, SNs were available to provide expert guidance to site investigators and coordinators who did not know how to navigate institutional IRB reliance requirements, site-specific consent preparation, and subcontract execution with institutional personnel.

Recently, the National Cancer Institute (NCI) announced a target site activation timeline of 90 days for its investigators [20], and many NIH institutes now require performance milestones for start-up. Our analysis demonstrates that, when using the three ASU elements, sites were more likely to activate within or more closely to a prescribed 90-day timeline. This suggests a formal start-up plan, such as ASU, may be helpful in improving site start-up timelines.

## Limitations

Other than tracking how completely the three ASU elements were applied to each trial, specific reasons why milestones were met at a faster or slower pace per site were not captured, limiting insight as to whether there were trends in this convenience sample that could predict site start-up performance. The major challenge to meeting shorter start-up timelines is managing unexpected events. Even the best planned and managed start-up can be derailed by losses in personnel, delayed approvals and clearances, or data trust and technology complications. Similarly, trial design, such as a complex protocol or ethical issues, could require multiple ancillary reviews or multiple rounds of a convened IRB and negatively impact start-up timelines. These events and others may occur at the central level, affecting all site activations, or at a site level as an isolated bottleneck. At the sites, the loss of personnel, whether temporary or permanent, will alter performance. Four non-COVID treatment trials activated during the COVID-19 pandemic (Trials A2, B1, C1, and C2) may have been negatively impacted by the pandemic when COVID treatment trials were prioritized at institutional levels and workplace limitations were placed on trial personnel; however, our sample was too small to determine if there was a COVID-19 period effect on non-COVID-19 trials or to perform a formal analysis of the relatedness of having personnel in place, or looking at the impact of protocol complexities or other factors on start-up performance.

Personnel in place at coordinating centers may be another limitation to implementing an ASU strategy. Our convenience sample may not fully represent or reflect the personnel capacity and electronic platform capabilities of other coordinating centers, which could lessen the generalizability of our ASU management strategy in some settings. Scalability at academic coordinating centers can be accomplished by determining the number of SNs needed based not on the number of studies per year but on the number of sites to be managed in any given month of the year. The target start-up period is approximately one quarter of a year (90 days). At a ratio of 25 sites per navigator, a single SN can effectively manage up to 100 sites over a year, whether all in one trial or distributed among multiple trials. A coordinating center will need the funding and resources to scale up personnel accordingly, but with the ASU’s protocolized work sequences and leaner parallel pathways, experienced AROs and CROs should be able to retool their workflow to revise the order and pace at which sites are activated, freeing up effort for the next wave of sites.

Even though the two COVID-19 treatment trials were started during unprecedented times, these two trials signaled that a significant amount of time can be saved when research teams and institutional support units respond to trial timelines on a priority basis. This fast pace will not become the new norm, as it is unsustainable [21,22]; however, the lessons learned from these studies reinforce that both research teams and institutions can mobilize much faster than the current norm when needed.

A limitation of the analysis is that site-level metrics were assumed to be independent between trials. We note that rarely was there a common site *investigator/team* between trials: the two COVID-19 trials were intentionally offered together to investigators in the interest of enrolling from the same participant pool. Otherwise, only three of 258 site teams participated in two trials (Figure 3, Trials C3 and C4). Each trial faced challenges unique to that trial, and thus site performance was subject to specific trial-wide effects. The results of analyses shown in Tables 2 and 3 were adjusted for trial-specific effects by including random intercepts. The inclusion of random intercepts allowed the site-level outcomes to be modeled as correlated within a trial.

There may be economies of learning once a site team learns the method; however, we have not surveyed to explore the endurance of these work habit changes. After one or two trials, experienced site teams may require less oversight, and specific institutions may have faster start-up records after multiple study teams that go through an ASU program with their contract offices and local IRB staff. Site teams were exposed to predefined benchmarks and expectations and observed that better management could improve timelines and foster a more efficient work environment. We hope these practices will be enduring at the sites, but endurance was not tested in this limited sample. But we also suspect that trials will be better served when trial leaders rely on the use of performance expectations (i.e., a predetermined workflow with standardized pathways and expected milestone completion times) and personnel effort dedicated to managing performance during the trial start-up phase.

## Conclusion

The ASU strategy addresses a top priority in eliminating multicenter clinical trial roadblocks. Strategic approaches to streamlining start-up tasks, persistent site management, and benchmarking milestones may help improve timelines. The accelerated start-up (ASU) management strategy used site navigators and the automated programming of tasks to target the order and pace in which start-up tasks should occur and to track progress and manage resources. These exploratory findings need to be further tested. It is our hope that these or similar practices will be embraced by more trial sponsors; however, systematic changes will be needed both centrally and at the sites to get to an overall start-up goal of 90 days or less as the performance standard for multicenter clinical trials.

## Supporting information

10.1017/cts.2025.10180.sm001 Hillery et al. supplementary material Hillery et al. supplementary material

## Supplementary material

The supplementary material for this article can be found at https://doi.org/10.1017/cts.2025.10180.
