# Supplementary material for: Accelerating start-up cycles in investigator-initiated multicenter clinical trials
Source: J Clin Transl Sci. 2025 Oct 24;9(1):e249. doi: 10.1017/cts.2025.10180 (PMC12695510; doi:10.1017/cts.2025.10180)
Supplement: Hillery et al. supplementary material [file S2059866125101805sup001.docx]

**Supplemental Material for “Accelerating Start-up Cycles in Investigator-Initiated Multicenter Clinical Trials”**

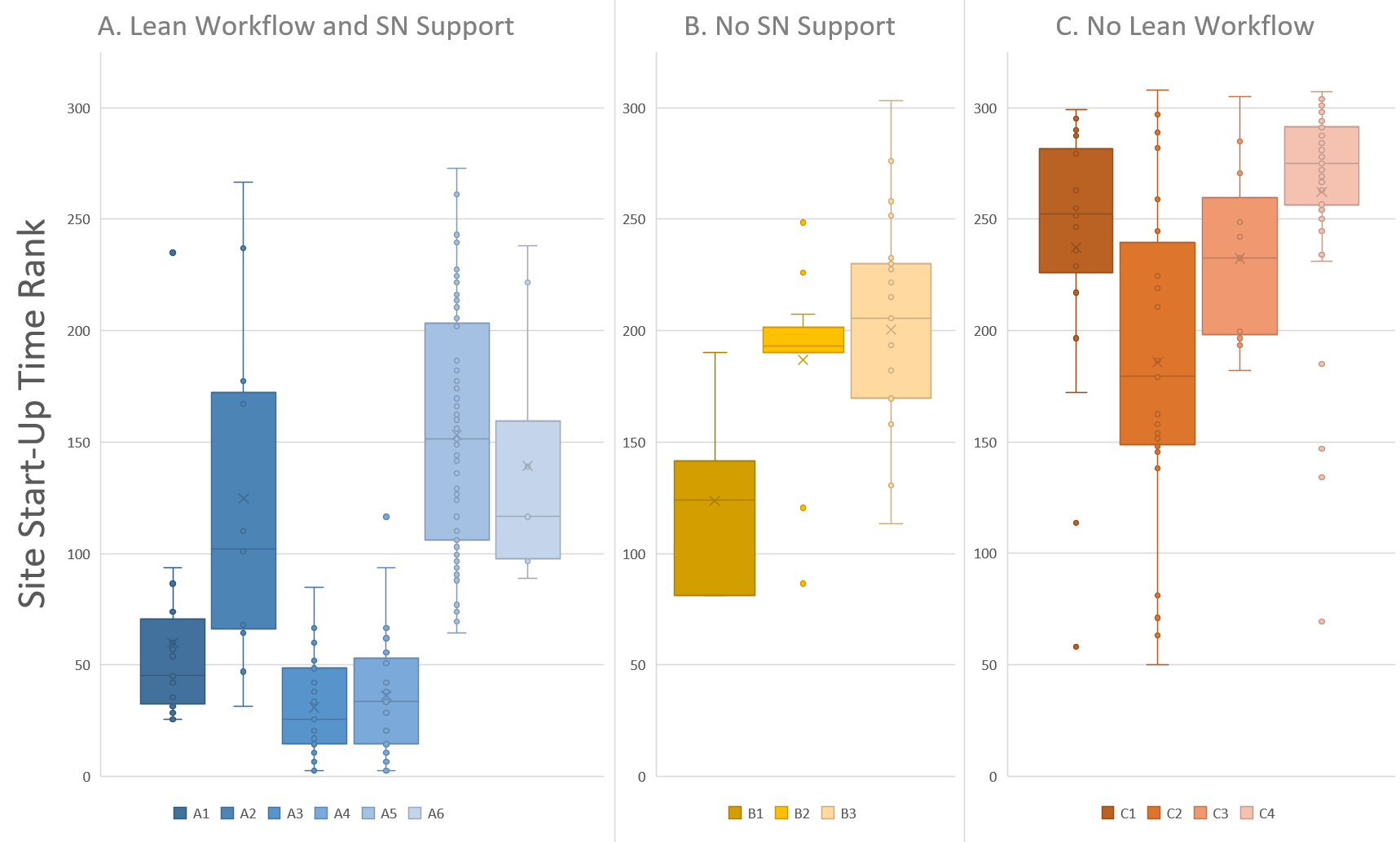


**Supplemental Figure 1.** Visual display of trial sites’ ranks, as ranked from fastest to slowest in start-up performance across 13 trials (n=308) with the COVID-19 trials highlighted. While some motivated sites in Panel C performed well, the majority performed slower than the sites in the other Panels. This figure supports Table 3 in the main article Linear Mixed Model (LMM) analyses being based on the ranks rather than directly on the number of days to reach the ASU goals (See main article, Figure 3.)

**SENSITIVITY ANALYSIS**:

**Sensitivity Analysis Methods:** Analysis was conducted using many identical methods as the primary manuscript analysis, but with the two COVID-19 trials (A3 and A4) removed. The generalized linear mixed models (GLMMs) for the odds of reaching the milestone benchmark were re-estimated with these two trials removed. Removal of these trials required re-ranking the sites from the remaining trials, and then the linear mixed models (LMMs) for the ranked data were re-estimated also. For the primary manuscript analyses, we also fitted Cox frailty models to assess for differences in time-to-event outcomes between combinations while allowing for within-study clustering of site-level performance. However, we do not report the Cox results for the primary manuscript analyses because of concerns that the proportional hazards assumption would not be reasonable. Consequently, we did not refit the Cox models after excluding the two COVID-19 trials.

**Sensitivity Analysis Results:** As with the primary analysis, across all seven metrics, Panel A had the highest overall percentage of sites meeting the timeline goal (Supplemental Table 1). There were fewer significant differences in the sensitivity analysis excluding the two COVID-19 trials. However, two outcomes retained statistical significance: sIRB submission (for the GLMM and ranked time LMM) and site activation (for the ranked time LMM). The other metrics all had estimates in the direction of Panel A performing the best but did not reach statistical significance. For two outcomes, completion of all study-specific training requirements and activation for enrollment, the GLMM estimation had convergence problems (note that at least one of the panels had 0 sites meet the timeline goal).

**Supplemental Table 1 (Analogue to Table 2) Sensitivity Analysis. Excluding Two COVID-19 Treatment Trials,** Percent of Site Teams Achieving Seven Key Pre-Determined Benchmarks, by ABC Combination, and the Odds of Executing Benchmarks (Dichotomized Success/Failure) Based on Generalized Linear Mixed Models (GLMMs).

|  |  | **Activity Benchmark Achieved (%)** | | |  | **Odds Ratio (95% CI)** | | |  |
| --- | --- | --- | --- | --- | --- | --- | --- | --- | --- |
| **Start-Up Activity** | **Predetermined Benchmark (# Days from “Go Live”)** | **Panel A**  **110 Sites**  *(Lean Workflow & SN Support)* | **Panel B**  **52 Sites**  *(No SN Support)* | **Panel C**  **96 Sites**  *(No Lean Workflow)* |  | **Panel A**  **110 Sites**  *(Lean Workflow & SN Support)* | **Panel B**  **52 Sites**  *(No SN Support)* | **Panel C**  **96 Sites**  *(No Lean Workflow)* | **P-value^1^** |
| sIRB Submission | 40 | 39% | 6% | 7% |  | Reference | 0.11 (0.01, 0.78) | 0.11 (0.02, 0.52) | 0.02 |
| Contract Partial Execution | 49 | 58% | 31% | 31% |  | Reference | 0.28 (0.05, 1.54) | 0.41 (0.09, 2.08) | 0.28 |
| Delegation of Responsibility Log | 49 | 29% | 2% | 6% |  | Reference | 0.01 (0.00, 2.30) | 0.02 (0.00, 1.84) | 0.12 |
| Regulatory Documents | 56 | 63% | 4% | 28% |  | Reference | 0.01 (0.00, 1.35) | 0.33 (0.00, 48.8) | 0.17 |
| Personnel Regulatory Documents | 56 | 64% | 4% | 21% |  | Reference | 0.01 (0.00, 1.12) | 0.28 (0.00, 18.71) | 0.15 |
| Training & Certificates | 77 | 17% | 0% | 2% |  | Not reported – convergence issues in model estimation | | |  |
| Activation to Enrollment Phase | 90 | 11% | 0% | 0% |  | Not reported – convergence issues in model estimation | | |  |

Abbreviations: CI, confidence interval; Q, quartile; sIRB, single institutional review board; SN, Site navigator.
Note: Excludes sites with a negative time-to-event duration or with missing dates such that a duration could not be calculated. Column header site counts reflect the number of sites activated. Denominator per metric may be slightly different.
^1^P-values from logistic regression models with random intercepts for the trial.

**Supplemental Table 2 (Analogue to Table 3) Sensitivity Analysis.** Organized by Figure 3 ABC Combinations, Analyses of Site Team Time to Completion of Seven Key Activities Based on Linear Mixed Models (LMMs) with the COVID-19 Trials omitted for sensitivity analysis. (See also Supplemental Figure 2 for depiction of site start-up time ranks for the 11 trials demonstrating the improvement in meeting LMM assumptions by using ranks of the data instead of the original data.)

|  | **Median (Q1, Q3), in Days** | | |  |
| --- | --- | --- | --- | --- |
| **Site Start-Up Activity** | **Panel A Sites**  **Lean Workflow and SN Support** | **Panel B Sites**  **No SN Support** | **Panel C Sites**  **No Lean Workflow** | **P-value^1^** |
| sIRB Submission | 48 (32, 92) | 91 (63, 115) | 96 (70, 190) | 0.02 |
| Contract Partial Execution | 43 (23, 71) | 77 (46, 106) | 89 (37, 147) | 0.44 |
| Finalized and Signed Delegation of Responsibility Log | 62 (47, 89) | 112 (89, 139) | 211 (84, 296) | 0.09 |
| Upload of All Site-Wide Regulatory Documents (Protocol Signature Page, Signed Investigator Brochure, etc.) | 50 (25, 69) | 126 (96, 150) | 108 (54, 235) | 0.27 |
| Upload of All Site Team Regulatory Documents (CVs, Human Subjects Training, Med./Prof. Licenses) | 50 (38, 70) | 119 (89, 148) | 154 (64, 243) | 0.16 |
| Completion of All Study-Specific Training Requirements | 141 (115, 171) | 152 (133, 199) | 219 (123, 312) | 0.12 |
| Activation to Enrollment | 150 (131, 196) | 191 (153, 217) | 277 (211, 347) | 0.01 |

^1^ P-value from linear mixed model using the ranked event duration as the outcome with random intercepts for trial.

SN, site navigator; sIRB, single institutional review board; Q, quartile; CVs, curriculum vitae; Med., medical; prof., professional

Note: Excludes sites with a negative time-to-event duration or with missing dates such that a duration could not be calculated.


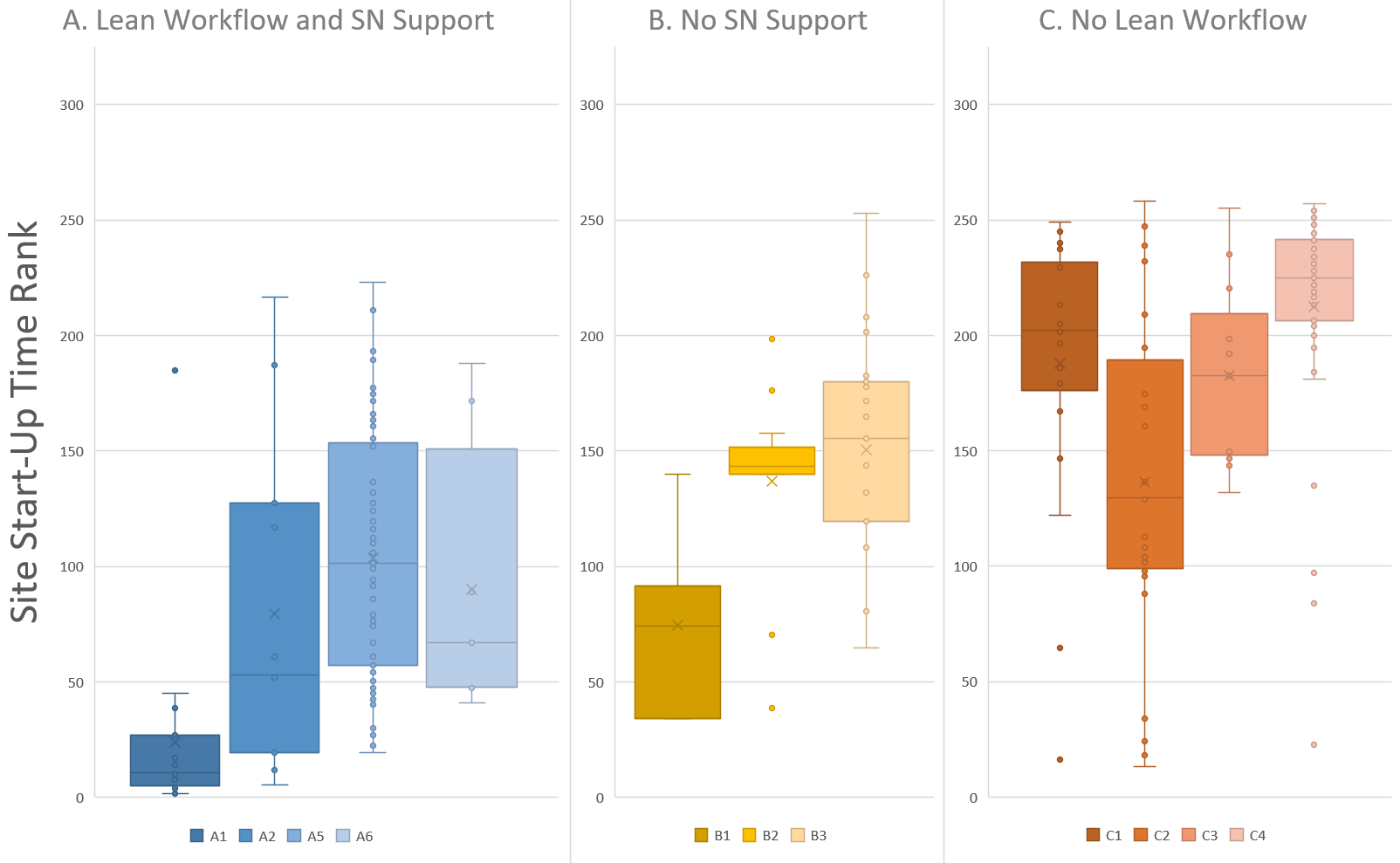


**Supplemental Figure 2:** Visual display of trial sites’ ranks, as ranked from fastest to slowest in start-up performance across 11 trials (COVID-19 Studies A3 and A4 removed). While some motivated sites in Panel C performed well, the majority performed slower than the sites in the other Panels. This figure supports Supplemental Table 2 Linear Mixed Model (LMM) analyses being based on the ranks rather than directly on the number of days to reach the ASU goals.
